# Sinus pilonidal

**DOI:** 10.11604/pamj.2013.15.20.2803

**Published:** 2013-05-10

**Authors:** Wafae Raffas, Badredine Hassam

**Affiliations:** 1 Service de Dermatologie, CHU Ibn Sina, Université Med V, Souissi, Rabat, Maroc

**Keywords:** Sinus pilonidal, hypoderme, hirsutisme, pilonidal sinus, hypodermis, hirsutism

## Image en médicine

La maladie pilonidale (kyste ou sinus pilonidal) est une pathologie fréquente et récidivante l'adulte jeune avec une prédominance masculine (75%). Il s'agit d'une cavité pseudokystique contenant des poils, siégeant le plus souvent dans l'hypoderme de la région sacrococcygienne. Elle serait la conséquence d'une réaction inflammatoire suite à la pénétration de poils dans la peau. Le phototype clair, la pilosité importante, l'obésité et les frottements répétés sont autant de facteurs favorisants. La maladie est asymptomatique jusqu'à l'infection avec tableau d'abcès aigu, de fistule(s) donnant issue à des sérosités ou du pus, ou de tuméfaction non-fistulisée. Le traitement s'adresse aux formes symptomatiques; il est chirurgical reposant sur un drainage associé à un curetage en cas d'abcès aigu, et sur des excisions plus ou moins larges suivies d'une cicatrisation dirigée, de sutures ou de plasties en cas de suppurations chroniques. Nous rapportons le cas d'une jeune fille de 22ans, sans antécédents notables, qui consultait pour une formation tumorale de la région sacrée évoluant depuis 4mois augmentant de volume et saignant au contact. À l'examen, elle présentait à la partie supérieure du pli interfessier, une petite tumeur pédiculée, de couleur rose, à surface érosive sensible à la palpation. L'examen retrouvait par ailleurs une surcharge pondérale et un hirsutisme à début péripubertaire. Plusieurs diagnostics étaient évoqués notamment un botriomycome, un mélanome achromique, ou fibrome traumatisé. La biopsie-exérèse du nodule était réalisée dont l'étude histologique révélait un kyste pilonidal. La patiente n'a pas présenté de récidive durant la période de suivi d'une année.

**Figure 1:**
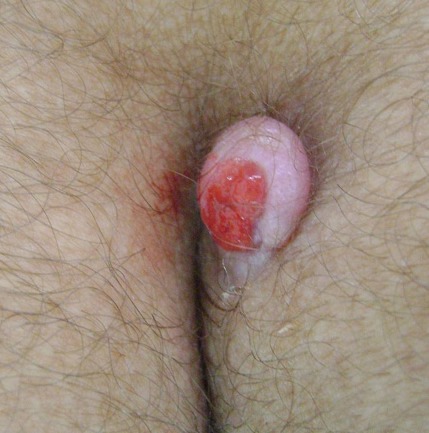
Tumeur ovalaire de 1,5cm x 2cm, pédiculée, jouxtant la partie supérieure du pli interfessier du côté gauche, de couleur rose à surface érodée

